# Supplementary material for: Resolving mechanisms of immune‐mediated disease in primary CD4 T cells
Source: EMBO Mol Med. 2020 Apr 1;12(5):e12112. doi: 10.15252/emmm.202012112 (PMC7207160; doi:10.15252/emmm.202012112)
Supplement: Supplementary file 1 — Appendix [file EMMM-12-e12112-s001.pdf]

Appendix

Resolving mechanisms of immune-mediated disease in primary CD4 T cells

Authors: Bourges C, Groff AF, Burren OS, Gerhardinger C, Mattioli K, Hutchinson A, Hu T, Anand T, Epping MW, Wallace C, Smith KGC, Rinn JL, Lee JC*

Contents

- **Appendix Table S1**. Summary of tiling analysis.
- **Appendix Table S2.** Primer sequences
- **Appendix Table S3.** gRNA sequences
- **Appendix Table S4.** Exact P values
- **Appendix Figure S1**. Minimal promoter-based MPRA does not work in primary CD4 T cells, but can be successfully adapted using a stronger promoter.
- **Appendix Figure S2**. Scaled Manhattan plots for 14 autoimmune disease associated loci – based on MPRA data from resting CD4 T cells.
- **Appendix Figure S3**. Scaled Manhattan plots for 14 autoimmune disease associated loci – based on MPRA data from stimulated CD4 T cells.
- **Appendix Figure S4**. rs6927172 lies in a highly conserved locus and is predicted to disrupt NF-κB binding.
- **Appendix Figure S5**. rs6927172 disrupts a *TNFAIP3* super-enhancer at 6q23.
- **Appendix Figure S6**. CRISPR-Cas9 editing in resting primary CD4 T cells.

Chr.	Haplotype co-ordinates (hg19)	Associated disease(s)	Driver elements in resting CD4 T cells	Driver elements in stimulated CD4 T cells	Driver elements (bp)
1p34	1:38614767-38644961	RA			294
2p15	2:62551372-62585241	AS, Ps			557
2p24	2:12632889-12648793	ATD			310
3p24	3:28068294-28079185	MS			121
4q32	4:166558881-166575539	T1D			336
5p13	5:40522112-40619865	AS			766
6p23	6:14711861-14734441	UC, CD, MS			559
6q23	6:137959135-138006604	RA, CeD, UC, CD, SLE, T1D			479
8q24	8:130602181-130624205	UC, CD			293
8q24	8:128187774-128207238	MS			32
11q21	11:95311160-95320908	ATD, vitiligo			49
14q32	14:98485011-98499051	T1D			261
21q21	21:16804230-16828335	UC, CD			212
21q22	21:40463183-40468938	AS, PSC, UC, CD			82
21q22	21:36421330-36423329	Positive control 1			49
1q31	1:198626200-198628199	Positive control 2			81
4p15	4:29562525-29564524	Negative control 1			0
4p15	4:34780413-34782412	Negative control 2			0

Appendix Table S1. Summary of tiling analysis.

Summary results from tiling analysis in resting and stimulated CD4 T cells using the *sharpr2* package

Filled green boxes indicate the presence of high-resolution driver elements with significant regulatory activity (FWER $P < 0.05$) within the genomic sequence of the disease-associated haplotype. Filled grey boxes indicate that no high-resolution driver elements were identified in the region.

Driver elements (bp) indicates the total number of bases within the disease-associated region that were identified as high-resolution driver elements in either resting or stimulated T cells.

Chr., chromosome; AS, Ankylosing Spondylitis; Ps, Psoriasis; PSC, Primary Sclerosing Cholangitis; UC, ulcerative colitis; CD, Crohn's disease; RA, rheumatoid arthritis; CeD, coeliac disease; SLE, Systemic Lupus Erythematosus; T1D, Type 1 Diabetes; ATD, autoimmune thyroid disease; MS, multiple sclerosis.

Target	Sequence
Oligo-pool amplification	**F:** GCTAAGGGCCTAACTGGCCGCTTCACTG<br>**R:** GTTTAAGGCCTCCGAGGCCGACGCTCTTC
MPRA library prep	**F:** CAAGCAGAAGACGGCATACGAGATNNNNNNGTGACTGGAGTTCAGACGTGTGCTCTTCCGATCTAACGAGAAGCGCGATCACA<br>**R:** AATGATACGGCGACCACCGAGATCTACACTCTTTCCCTACACGACGCTCTTCCGATCT
TurboGFP	**F:** AGGACAGCGTGATCTTCACC<br>**R:** CTTGAAGTGCATGTGGCTGT
EGFP	**F:** GCTACCCCGACCACATGAAG<br>**R:** TCTTGTAGTTGCCGTCGTCC
eRNA PCR_1	**F:** CCCTGGGAGCCTGTGAAAAT<br>**R:** AACAGGGAAGCCAGAGATGC
eRNA PCR_2	**F:** CACACGCCAGAAACATCTGC<br>**R:** TGACTGTGATTTCTCCCTGAGG
rs1988588 SDM	**F:** CAACAGAGCGAGACTCCGTC<br>**R:** CCCAGGCTGGAGTGCAG
rs3902659 SDM	**F:** GCGGAGCTTGCAGTGAGC<br>**R:** CTCCTGGGTTCACGCCAT
Firefly luciferase	**F:** GCTCAGCAAGGAGGTAGGTG<br>**R:** TCTTACCGGTGTCCAAGTCC
Renilla luciferase	**F:** ATCGGACCCAGGATTCTTTT<br>**R:** ACTCGCTCAACGAACGATTT
TNFAIP3	**F:** AGGTTCCAATTTCGCCCCTT<br>**R:** GAACAGCTCGGATTTCAGGC
OLIG3	**F:** ATTTCCCGCCTAAAGCCTCC<br>**R:** GTGGACGAGACCGAGTTGAG
IL20RA	**F:** ATGGGCAAAAGAAATGGCTG<br>**R:** GGTGGGCCAATTTGTGTTTCT
IL22RA2	**F:** TGGTGTAGCAGGAACTCAGTC<br>**R:** CTGCTGTTGCCAGTAAGTGC
IFNGR1	**F:** GAAGTGACGTAAGGCCGGG<br>**R:** TAGTTGGTGTAGGCACTGAGGA
PERP	**F:** TGTGGTGGAAATGCTCCCAA<br>**R:** TACCCCACGCGTACTCCAT
β-Actin	**F:** GAGCATCCCCCAAAGTTCA<br>**R:** AGAGAAGTGGGGTGGCTTTT
HPRT (T7EI)	**F:** AAGAATGTTGTGATAAAAGGTGATGCT<br>**R:** ACACATCCATGGGACTTCTGCCTC
CXCR4 (ICE)	**F:** GACGCCAACATAGACCACCT<br>**R:** TGCTTGCTGAATTGGAAGTG
rs6927172 locus (ICE)	**F:** GTAGTACCCTGGGAGCCTGT<br>**R:** GTCCTGAGAAGCAGCTTGGT
rs35926684 locus (ICE)	**F:** GGTGAGGGAAAATCAGACAGA<br>**R:** GCAGGAATCAGCCATTTCTC
rs17264332 locus (ICE)	**F:** TCACGAGAATGCCTGCATAG<br>**R:** TCCCTGATCACATCACTCCA
rs11757201 locus (ICE)	**F:** GGGTCACTAGTGGAGCCAAA<br>**R:** CCCCCTCAAAAAGTGGACAAA
rs6920220 locus (ICE)	**F:** CCTTGAGCCACCTGCTTTAG<br>**R:** AATGCTTGGACCTTGATTGG
TNFAIP3 gRNA1 (ICE)	**F:** AAACACTGGGGTTTCCTGCA<br>**R:** TTACGGGCCAGAGAAGGGTA
TNFAIP3 gRNA2 (ICE)	**F:** CTCTTCATCACAGGCCTGCA<br>**R:** ATCCAAGTGCCTTGTGTGGT

Appendix Table S2. Primer sequences

NNNNNN in MPRA library prep F primer represents sequencing index. SDM, site-directed mutagenesis; T7EI, T7 endonuclease I assay; ICE, Inference of CRISPR Edits

Name	gRNA sequence (excluding PAM)
HPRT crRNA	*Alt-R CRISPR-Cas9 Positive Control Human HPRT, IDT*
CXCR4 crRNA	GAAGCGTGATGACAAAGAGG
D_5'_rs6927172 crRNA	ATATTTCGGAGCTAATCAAG
F_5'_rs6927172 crRNA	TCAAGTGGCAATGTCAATGG
B_3'_rs6927172 crRNA	GATGGGAATTAAAGTTGACC
H_3'_rs6927172 crRNA	TTCTGCCACTTAGTCATGAT
5'_rs17264332 crRNA	GTACTTAATAAAATAACAGT
3'_rs17264332 crRNA	ACTTCAATTGCTCAACAACA
5'_rs11757201 crRNA	TTTGTTATACTTTAAGTTCT
3'_rs11757201 crRNA	CACCTATGAGTGAGAACATG
5'_rs35926684 crRNA	AACATTACTACATTGAAGTG
3'_rs35926684 crRNA	TTGATTTGATTTGATATGCA
5'_rs6920220 crRNA	AAGGTTTTGAGACATTGCTA
3'_rs6920220 crRNA	GATATGGTTCTGTAGAACAA
TNFAIP3 crRNA 1	CTTGTGGCGCTGAAAACGAA
TNFAIP3 crRNA 2	TATGCCATGAGTGCTCAGAG
Negative control 1 crRNA	*Alt-R CRISPR-Cas9 Negative Control crRNA #1, IDT*
Negative control 3 crRNA	*Alt-R CRISPR-Cas9 Negative Control crRNA #3, IDT*
tracrRNA	*Alt-R CRISPR-Cas9 tracrRNA, ATTO™ 550, IDT*

Appendix Table S3. gRNA sequences

gRNA sequence (or source if commercially available) shown.

Fig 2B	P Value
rs140522	0.000760
rs1893592	4.10E-11
rs1923187	7.49E-19
rs41268482	3.61E-12
rs5889371	0.065121
rs9283753	0.068112
rs9661285	0.027677
GATA1	0.008371
NF-κB	0.000570
RUNX1	1.31E-08
Neg. control	0.720853

Fig 3A	P Value (FDR-corr.)
rs1736137 (resting T cells)	8.21E-13
rs1736137 (stim. CD4 T cells)	1.76E-12

Fig 3B	P Value (FDR-corr.)
rs6759298	1.17E-12
rs13001372	0.856276
rs4672505	0.372014

Fig 4C	P Value (FDR-corr.)
rs6927172 (resting T cells)	2.13E-07
rs6927172 (stim. CD4 T cells)	2.81E-07

Fig 4E	P Value
Allele-specific NF-κB binding	0.0037

Fig 4F	P Value
Allele-specific expression (RNA)	0.0006

Fig 4I	P Value
TNFAIP3 eQTL CD4 T cells (active IBD)	0.04662

Fig 5D	P Value
Change in TNFAIP3 transcription vs non-targeting control	< 0.0001

Fig 5E	P Value
CD69 expression (ATTO+ vs ATTO- cells, DB gRNA combination)	0.0067
CD69 expression (ATTO+ vs ATTO- cells, DH gRNA combination)	0.0146
CD69 expression (ATTO+ vs ATTO- cells, FH gRNA combination)	0.0034

Fig 5F	P Value
Linear regression of increase in Phospho-IκBα vs editing efficiency	0.00099

Fig 5G	P Value
IFNγ: DB gRNA combination vs NTC	0.0057
IFNγ: DH gRNA combination vs NTC	0.0031
IFNγ: FH gRNA combination vs NTC	0.0056
IL-17: DB gRNA combination vs NTC	0.0136
IL-17: DH gRNA combination vs NTC	0.0248
IL-17: FH gRNA combination vs NTC	0.0164
IL-4: DB gRNA combination vs NTC	0.0075
IL-4: DH gRNA combination vs NTC	0.0078
IL-4: FH gRNA combination vs NTC	0.0089

Appendix Fig S4C	P Value
Allele-specific NF-κB binding to MPRA vector (major vs minor allele at rs6927172)	0.00013

Appendix Fig S6C	P Value
rs35926684 deletion: TNFAIP3 expression vs NTC	0.995
rs17264332 deletion: TNFAIP3 expression vs NTC	0.3621
rs11757201 deletion: TNFAIP3 expression vs NTC	0.6243
rs6920220 deletion: TNFAIP3 expression vs NTC	0.353
rs6927172 deletion: TNFAIP3 expression vs NTC	< 0.0001

Appendix Fig S6D	P Value
CD69: ETS2 gRNA1 vs NTC	0.0237
CD69: ETS2 gRNA2 vs NTC	0.0457

Appendix Fig S6D	P Value
IFNγ: ETS2 gRNA1 vs NTC	0.0135
IFNγ: ETS2 gRNA1 vs NTC	0.0491
IL-17: ETS2 gRNA1 vs NTC	0.0499
IL-17: ETS2 gRNA1 vs NTC	0.0447
IL-4: ETS2 gRNA1 vs NTC	0.0692
IL-4: ETS2 gRNA1 vs NTC	0.0447

Appendix Table S4. Exact P values

Exact P values or FDR-corrected P values (FDR-corr.) for all comparisons in manuscript

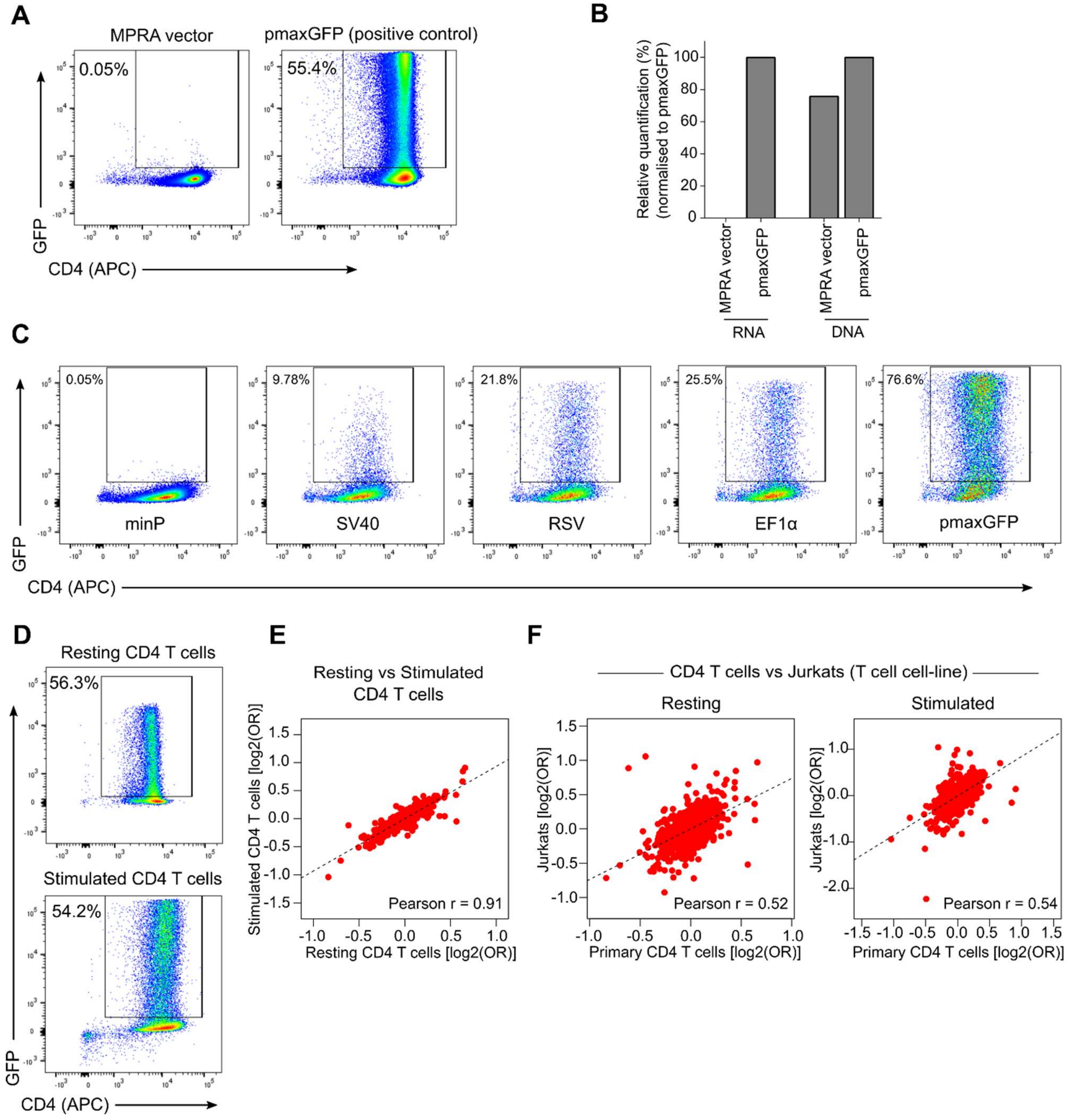

Appendix Figure S1. Adaptation of MPRA for use in primary CD4 T cells.

A Nucleofection of a minimal promoter-based MPRA vector into primary CD4 T cells does not lead to detectable GFP expression after 24 hours, unlike the positive control vector (pmaxGFP). **B** 24 hours after transfecting a minimal promoter-based MPRA vector into primary CD4 T cells, the vector can be recovered from the cells – confirming successful transfection – but no GFP RNA is detectable. Quantification by qPCR. **C** Flow cytometric assessment of the activity of a series of alternate promoters in primary CD4 T cells – all assayed 24 hours after transfection of 2µg vector into 5M CD4 T cells. **D** Representative plots of GFP expression 24 hours after nucleofecting an adapted MPRA vector (containing the RSV promoter, 5µg) into resting and stimulated primary CD4 T cells. **E** Comparison of SNP effects (log2(OR)) in resting and stimulated primary CD4 T cells. **F** Comparison of SNP effects (log2(OR)) in CD4 T cells and Jurkats (left panel, unstimulated; right panel, stimulated) revealing weaker correlation with several discordant effects.

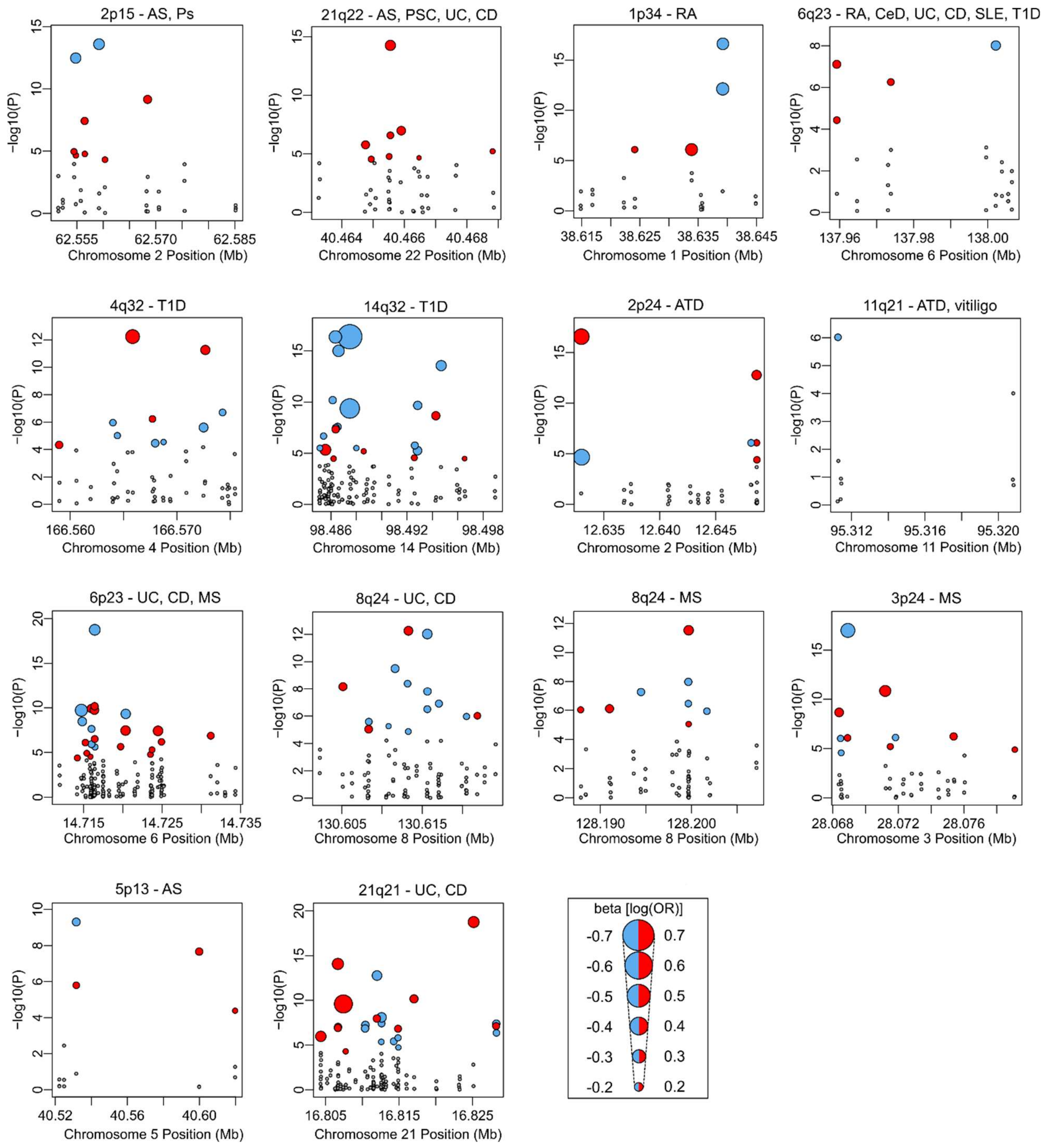

Appendix Figure S2. Scaled Manhattan plots for 14 autoimmune disease associated loci – based on MPRA data from resting CD4 T cells.

Scaled Manhattan plots of candidate SNPs in 14 autoimmune disease associated loci – based on expression-modulating effects in resting CD4 T cells. For SNP constructs with significant expression modulating effects (meta analysis $P < 0.05/970$) the size of each point is scaled to the effect size observed in the assay. The colour indicates the direction of the expression-modulating effect with respect to the risk allele. SNP constructs that did not pass this significance threshold are shown in grey. AS, Ankylosing Spondylitis; Ps, Psoriasis; PSC, Primary Sclerosing Cholangitis; UC, ulcerative colitis; CD, Crohn's disease; RA, rheumatoid arthritis; CeD, coeliac disease; SLE, Systemic Lupus Erythematosus; T1D, Type 1 Diabetes; ATD, autoimmune thyroid disease; MS, multiple sclerosis.

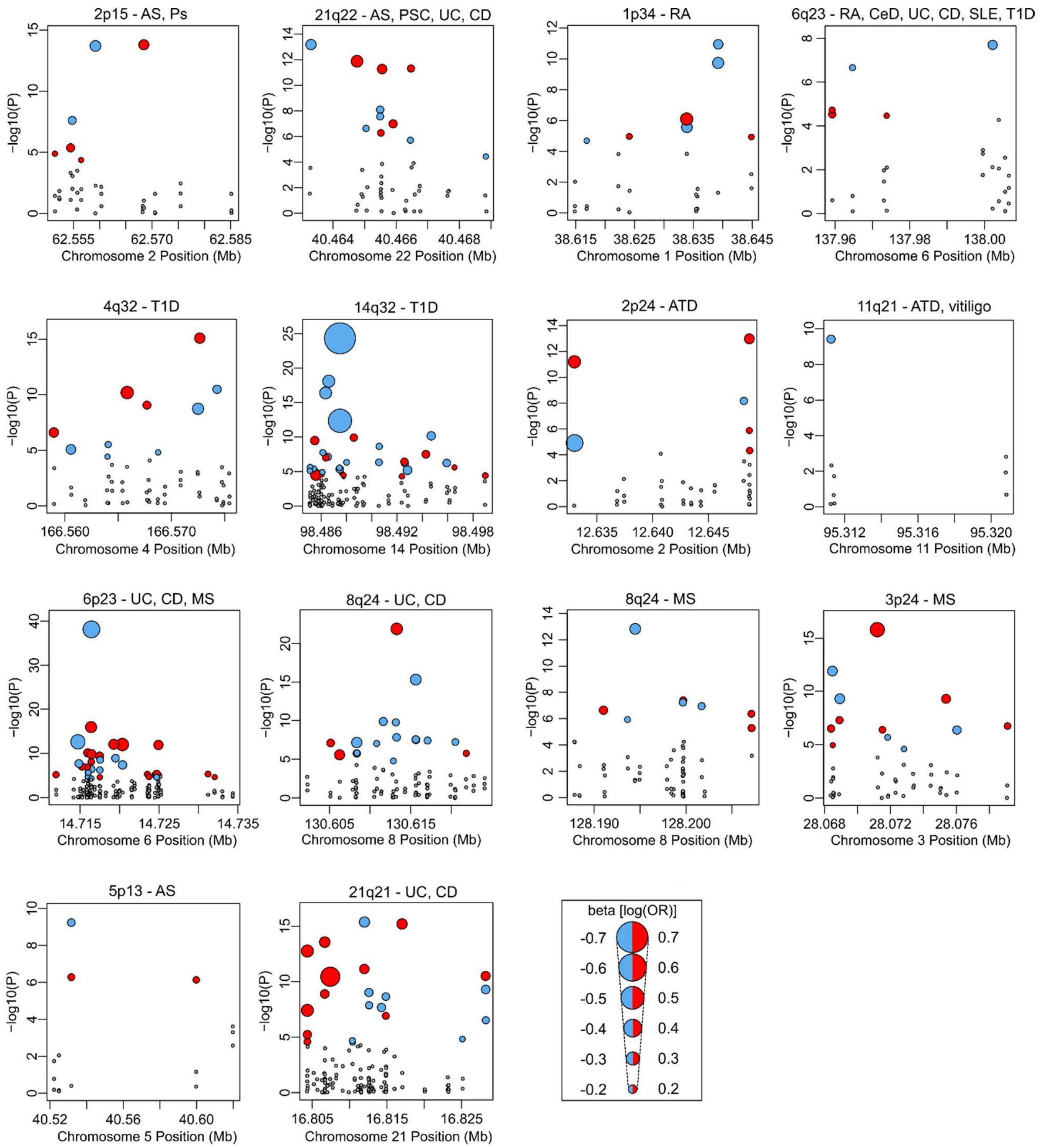

Appendix Figure S3. Scaled Manhattan plots for 14 autoimmune disease associated loci – based on MPRA data from stimulated CD4 T cells.

Scaled Manhattan plots of candidate SNPs in 14 autoimmune disease associated loci – based on expression-modulating effects in stimulated CD4 T cells. For SNP constructs with significant expression modulating effects (meta analysis $P < 0.05/970$) the size of each point is scaled to the effect size observed in the assay. The colour indicates the direction of the expression-modulating effect with respect to the risk allele. SNP constructs that did not pass this significance threshold are shown in grey. AS, Ankylosing Spondylitis; Ps, Psoriasis; PSC, Primary Sclerosing Cholangitis; UC, ulcerative colitis; CD, Crohn’s disease; RA, rheumatoid arthritis; CeD, coeliac disease; SLE, Systemic Lupus Erythematosus; T1D, Type 1 Diabetes; ATD, autoimmune thyroid disease; MS, multiple sclerosis.

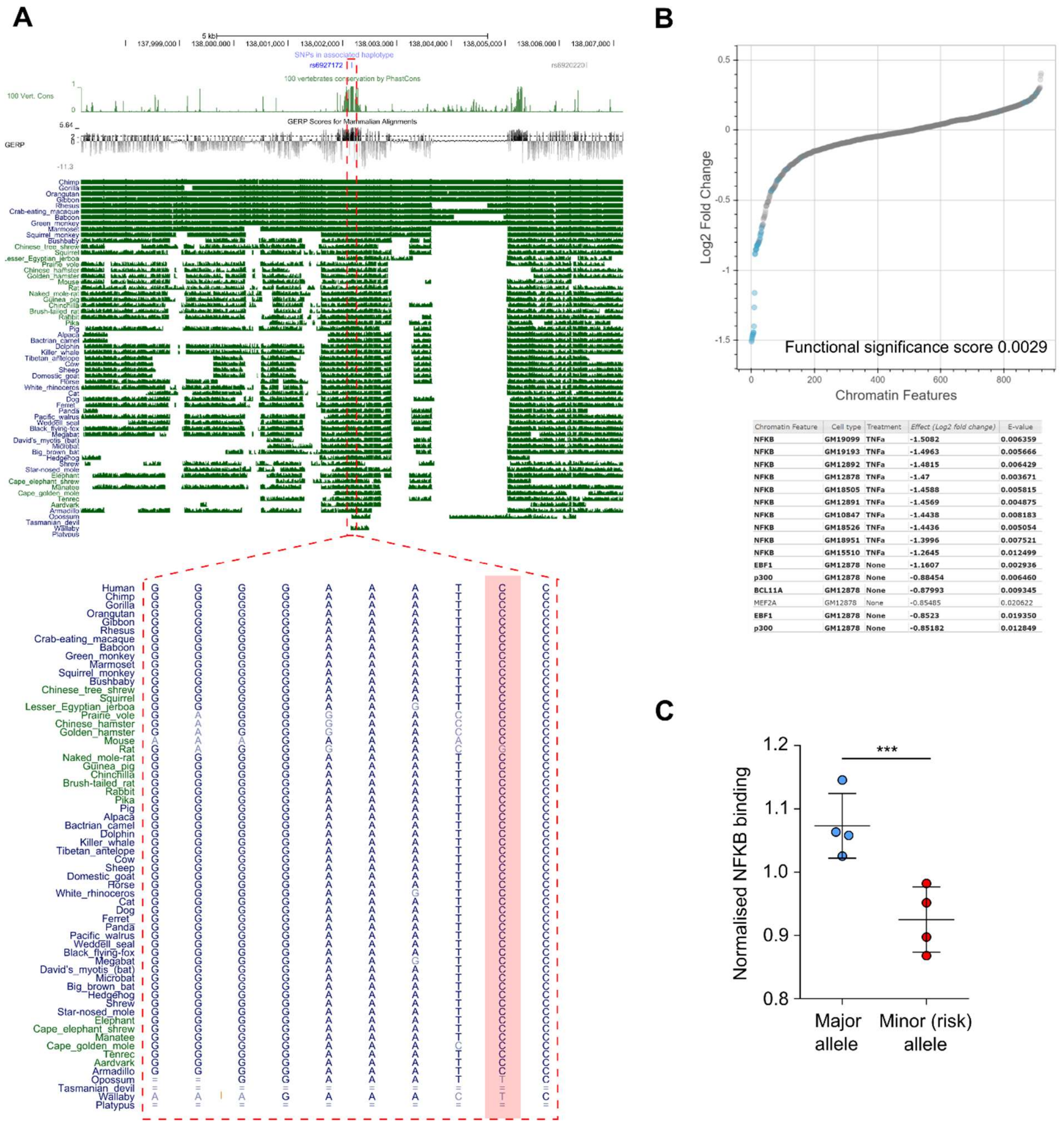

Chromatin Feature	Cell type	Treatment	Effect (Log2 fold change)	E-value
NFKB	GM19099	TNFa	-1.5082	0.006359
NFKB	GM19193	TNFa	-1.4963	0.005666
NFKB	GM12892	TNFa	-1.4815	0.006429
NFKB	GM12878	TNFa	-1.47	0.003671
NFKB	GM18505	TNFa	-1.4588	0.005815
NFKB	GM12891	TNFa	-1.4569	0.004875
NFKB	GM10847	TNFa	-1.4438	0.008183
NFKB	GM18526	TNFa	-1.4436	0.005054
NFKB	GM18951	TNFa	-1.3996	0.007521
NFKB	GM15510	TNFa	-1.2645	0.012499
EBF1	GM12878	None	-1.1607	0.002936
p300	GM12878	None	-0.88454	0.006460
BCL11A	GM12878	None	-0.87993	0.009345
MEF2A	GM12878	None	-0.85485	0.020622
EBF1	GM12878	None	-0.8523	0.019350
p300	GM12878	None	-0.85182	0.012849

Appendix Figure S4. rs6927172 lies in a highly conserved locus and is predicted to disrupt NF-κB binding.

A Three analyses of conservation: PhastCons, Genomic Evolutionary Rate Profiling (GERP) and Multiz all show that rs6927172 lies in a highly conserved region. Data from UCSC Genome Browser. rs6927172 highlighted in pink in lower panel. **B** DeepSEA analysis of candidate SNPs at 6q23 locus (using machine learning of regulatory sequence code from ENCODE chromatin-profiling data) predicts that rs6927172 is functionally significant with a significant effect on NF-κB binding. Inset table shows top results for rs6927172 ordered by effect size. **C** Following nucleofection of the MPRA vector library into primary CD4 T cells from 4 healthy individuals, cells were cross-linked and NF-κB immunoprecipitation was performed. Isolated plasmids were sequenced to assess for differential NF-κB binding. A SNP construct for rs6927172 showed significant allele-specific NF-κB-binding, with reduced binding to the risk allele containing vector.

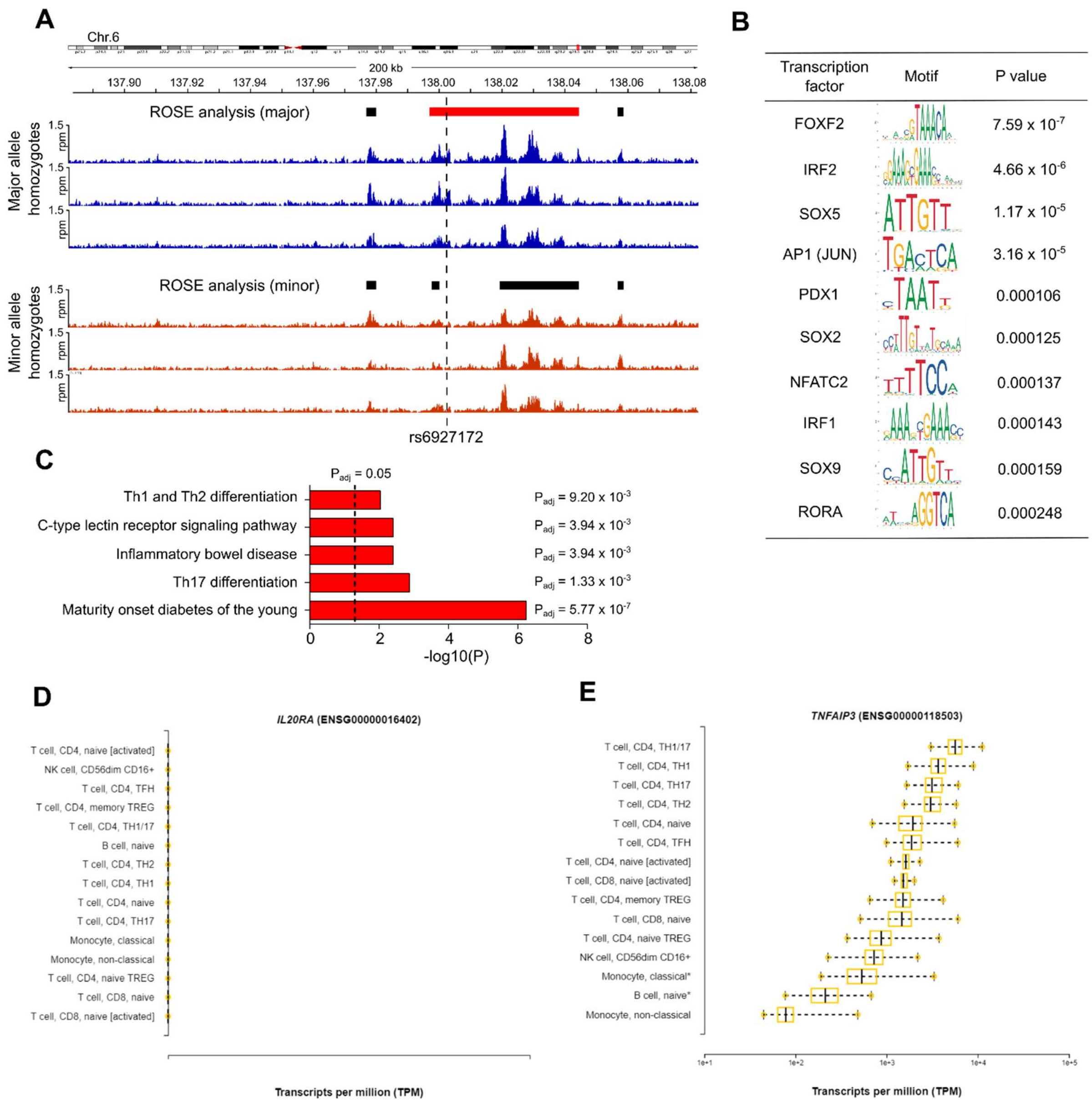

Appendix Figure S5. rs6927172 disrupts a *TNFAIP3* super-enhancer at 6q23.

A Normalised H3K27ac ChIP seq reads at the 6q23 locus in individual minor and major allele homozygotes at rs6927172. Bars indicate results from ROSE analysis (Ranking of Super-Enhancers, black = enhancer activity; red = super-enhancer). **B** The top 10 transcription factors whose binding motifs are over-represented within the constituent enhancer elements within the super-enhancer (compared to a background model based on all human promoters). Analysis performed using TRAP (TRanscription factor Affinity Prediction using Jaspar vertebrate matrices and Benjamini Hochberg correction for multiple testing). **C** Pathway analysis results (KEGG, FDR P < 0.01) using all 50 significantly-overrepresented transcription factors from TRAP analysis. Analysis performed using gProfiler. **D and E** Expression of *IL20RA* (**D**) and *TNFAIP3* (**E**) in primary immune cells from DICE database.

A

Nucleofection programme	Nucleofection buffer	Electroporation enhancer	Electropherogram	Editing efficiency (T7 endonuclease assay)
U014	Nucleofector solution for Human T Cells (Lonza)	-		21.4%
U014	1M solution (Chicaybam et al. 2013)	-		23.5%
U014	1M solution (Chicaybam et al. 2013)	+		25.8%
V024	Nucleofector solution for Human T Cells (Lonza)	-		35.7%
V024	1M solution (Chicaybam et al. 2013)	-		41.2%
V024	1M solution (Chicaybam et al. 2013)	+		45.5%

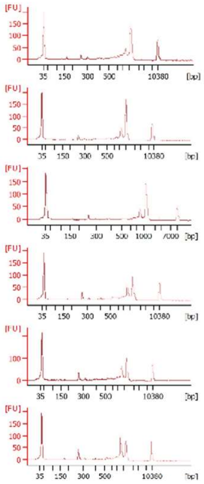

% editing efficiency = 100 x (1 - (1 - fraction cleaved)$^{1/2}$) where fraction cleaved = [digested products] / [digested products + undigested band]

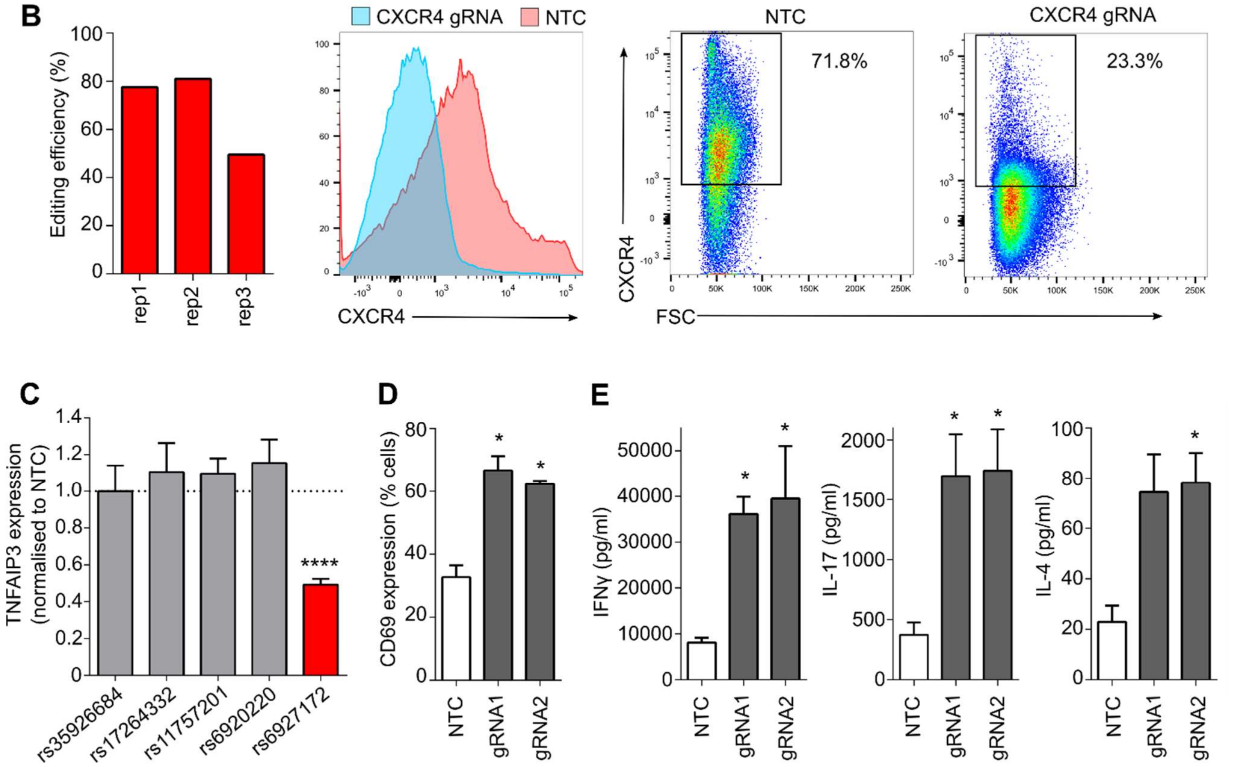

Appendix Figure S6. CRISPR-Cas9 editing in resting primary CD4 T cells.

A Optimisation of CRISPR editing in resting CD4 T cells using a Cas9 RNP containing positive control gRNA (targeting *HPRT*). On-target editing was assessed using a T7 Endonuclease assay. **B** Editing efficiency at the *CXCR4* locus assessed using ICE (left panel). Representative histograms and flow cytometry plots of CXCR4 expression on CD4 T cells following CRISPR editing (right panels). **C** *TNFAIP3* expression in EU-containing mRNA (EU added at time of stimulation) following individual deletions of candidate SNPs within the *TNFAIP3* super-enhancer (data from a minimum of 4 biological replicates). Mean indel rates: rs35926684, 55.0%; rs17264332, 54.2%; rs11757201, 52.0%; rs6920220, 72.1%; rs6927172, 64.5%). **D** Percentage of CD4 T cells expressing CD69, an activation marker, following CRISPR editing of *TNFAIP3* (n=4, paired *t*-test, one-tailed). **E** Secretion of IFNγ, IL-17A and IL-4 following CRISPR editing of *TNFAIP3* in activated CD4 T cells (n=4, paired *t*-test, one-tailed). Data represent mean +/- SEM. * $P < 0.05$; **** $P < 0.0001$.
